# Remote sensing of ice albedo using harmonized Landsat and Sentinel 2 datasets: validation

**DOI:** 10.1080/01431161.2023.2291000

**Published:** 2023-12-26

**Authors:** Shunan Feng, Joseph Mitchell Cook, Yukihiko Onuma, Kathrin Naegeli, Wenxia Tan, Alexandre Magno Anesio, Liane G. Benning, Martyn Tranter

**Affiliations:** a Department of Environmental Sciences, Aarhus University, Roskilde, Denmark; b Earth Observation Research Center, Japan Aerospace Exploration Agency (JAXA), Tsukuba, Japan; c Department of Geography, Remote Sensing Laboratories, University of Zurich, Zurich, Switzerland; d Key Laboratory for Geographical Process Analysis & Simulation of Hubei Province, College of Urban and Environmental Sciences, Central China Normal University, Wuhan, China; e State Key Laboratory of Geodesy and Earth’s Dynamics, Innovation Academy for Precision Measurement Science and Technology, CAS, Wuhan, China; f GFZ German Research Centre for Geosciences, Section Interface Geochemistry, Telegrafenberg, Germany; g Department of Earth Sciences, Free University of Berlin, Berlin, Germany

**Keywords:** Ice albedo, data harmonization, spatial window size, validation, arctic and alpine, Google Earth Engine

## Abstract

Albedo plays a key role in regulating the absorption of solar radiation within ice surfaces and hence strongly regulates the production of meltwater. A combination of Landsat and Sentinel 2 data provides the longest continuous medium resolution (10–30 m) earth surface observatory records. An albedo product (harmonized satellite albedo, hereafter HSA) has already been developed and validated for the Greenland Ice Sheet (GrIS), using harmonized Landsat 4–8 and Sentinel 2 datasets. In this paper, the HSA was validated for various Arctic and alpine glaciers and ice caps using *in situ* measurements. We determine the optimal spatial window size in point-to-pixel analysis, the best practices in evaluating remote sensing algorithms with groundtruth data, and cross sensor comparison of the Landsat 9 (L9) and Landsat 8 (L8) data. The impact of the spatial window size on measured ice surface homogeneity and albedo validation was analysed at both local and regional scales. Homogeneity statistics calculated from the grey-level co-occurrence matrix (GLCM) suggest that the ice surface becomes more homogeneous as the image resolution becomes coarser. The optimal spatial window size was found to be 90 m, based on maximizing the statistical and graphical measures while minimizing the root mean square error and bias. HSAs generally agree closely with *in situ* albedo measurements (e.g. Pearson’s R ranges from 0.68 to 0.92) across various Arctic and alpine glaciers and ice caps. Cross sensor differences between L9 and L8 are minor, and we suggest that no harmonization is necessary to add L9 to our HSA product.

## 1. Introduction

Albedo is a key component in glacier energy and surface mass balance (Ren et al. 2021; Van Angelen et al. 2012; Van Pelt et al. 2012, 2019; Zekollari and Huybrechts 2018), modulating the amount of solar radiation that is absorbed at the ice surface (Box et al. 2012; Irvine-Fynn et al. 2021; Van Den Broeke et al. 2008), and so is a primary control on glacier surface melt rates (Alexander et al. 2014; Box et al. 2012; Khan et al. 2015; Paul, Machguth, and Kääb 2005). For example, the recent reduction of surface ice albedo may account for 30%–60% of the total glacier melt in Tibetan Plateau (Zhang et al. 2021) and more locally on alpine glaciers (Naegeli et al. 2015). The collection and measurement of *in situ* albedo data is hampered by the remote and harsh nature of Arctic and alpine environments. It is also challenging to obtain spatially distributed ground-based glacier surface albedo (Brock, Willis, and Sharp 2000; Brock et al. 2007), particularly for larger ice masses, such as the Greenland Ice Sheet (GrIS). Further, the length of the melt season, the harsh climatic and ground conditions during both the spring thaw and the fall freeze up combine to bias ground measurements towards the more clement summer melt season.

**Figure A1.**
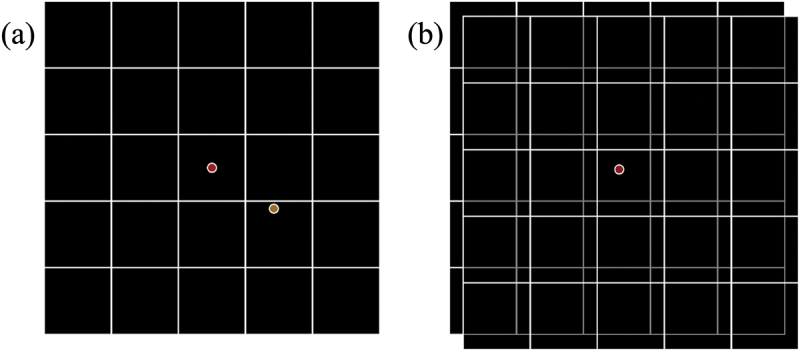
Conceptual diagram of point-to-pixel process. The scenario of moving sampling sites is illustrated in subfigure a and the influence of image co-registration error is shown in subfigure b.

Satellite imagery-derived albedo products, such as the Moderate Resolution Imaging Spectroradiometer (MODIS) MOD10A1 Terra Snow Cover Daily Global 500 m product (Hall, Riggs, and Salomonson 1995; Hall, Salomonson, and Riggs 2016; Hall et al. 2018; Hall, Riggs, and Salomonson 1995; Hall, Salomonson, and Riggs 2016) and the Global LAnd Surface Satellites (GLASS) albedo (Liu et al. 2013), allow rapid monitoring of ice albedo over large areas (Liang et al. 2003), and often over long periods of time. However, these datasets are not suitable for the complex morphologies, geographical settings and typical sizes (length scales are often 1–20 km) of alpine glaciers due to their coarse spatial resolution. The surface albedos derived from remote sensing imagery at medium resolution (10–30 m) provide valuable observations for the development of albedo parameterizations and glacier surface mass balance (SMB) models (Brock, Willis, and Sharp 2000; Knap, Reijmer, and Oerlemans 1996, 1999; Naegeli et al. 2017). However, the development and validation of remote sensing algorithms and/or models often requires linkage of point scale field measurements/simulations with pixel values measured from satellites (Liang 2001; Van der Meer 2012; Wood et al. 2012). This is often achieved by first minimizing image noise and pixel misregistration in the image collection by aggregating pixel values in an n × n window centred on the pixel (Figure A1) incorporating the ground sampling site (Kennedy, Yang, and Cohen 2010). This n×n window size usually produces an image with coarser spatial resolution in comparison to the ground sampling distance (Wu and Li 2009), and can introduce errors because the albedo of the window may differ from that of the ground measurement site. For example, Ryan et al. (2017) suggests that automatic weather station (AWS) measurements may overestimate albedo by 0.1 due to the discrepancy between the spatial resolution of the remote sensing data and the footprint of the AWS sensors. Therefore, it is very important to determine the appropriate spatial window size and assess its impact when validating the satellite-derived albedo.

**Figure 1.**
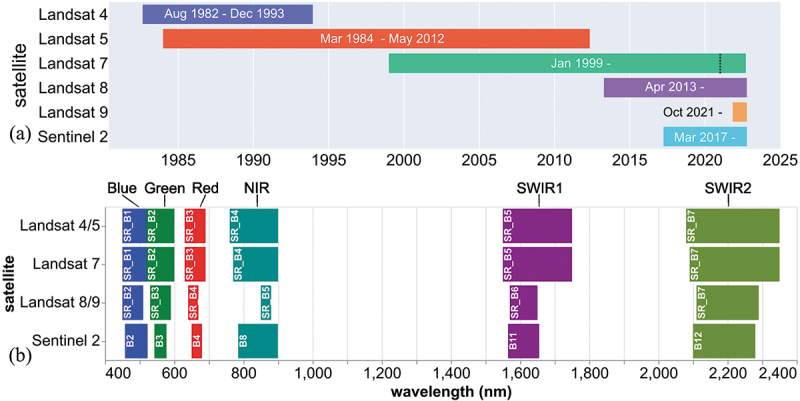
The timeline of data availability on Google Earth Engine (a) and the band designations of Landsat 4–9 and Sentinel 2 (b). The dashed line indicates the date when Landsat 7 (L7) data was excluded from this study due to the impact of orbit drift. The mission activities of L7 can be found at: https://www.usgs.gov/landsat-missions/landsat-7.

**Figure 2.**
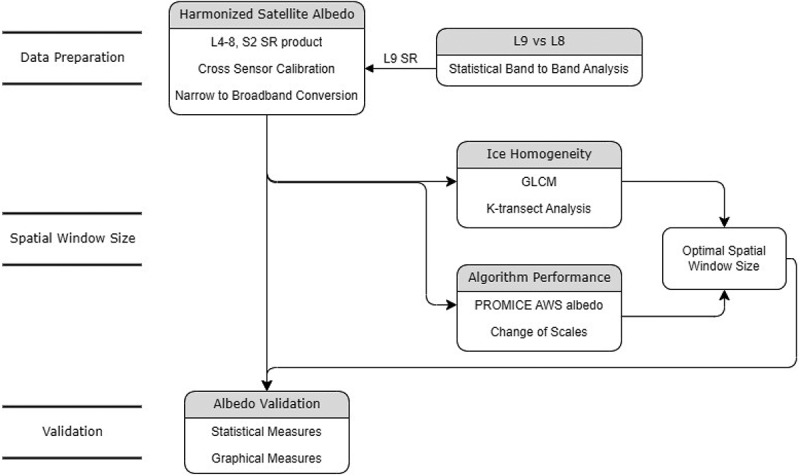
Flowchart of the albedo validation workflow.

Recently, we derived the broadband albedo (or harmonized satellite albedo, hereafter HSA) of the GrIS at 30 m resolution from a long time series (Figure 1a) of harmonized Landsat 4–8 and Sentinel 2 surface reflectance data (Feng et al. 2023). The derivation of HSA involves two steps (Figure 2): 1) cross sensor calibration, known as harmonization, and 2) narrow to broadband conversion. The albedo product has been validated by *in situ* measurements from the Program for Monitoring of the Greenland Ice Sheet (PROMICE) AWSs (Fausto et al. 2021; Van as and Fausto 2011). Further validation steps need to be undertaken to apply the albedo product to other glaciated areas because of the following limitations. First, the application of the HSA has only been validated for the GrIS. Second, the influence of the spatial window size has yet to be analysed systematically. Finally, a recent satellite change also requires incorporation into the albedo product to maintain the longevity of the time series. The Landsat 9 (L9) Operational Land Imager 2 (OLI-2) was designed as a ‘near clone’ of the Landsat 8 (L8) OLI for the visible to shortwave infrared bands (Figure 1b), and was successfully launched on 27 September 2021 (Masek et al. 2020; Wulder et al. 2022), and requires incorporation and cross sensor calibration within our glacier surface albedo product.

Here, we determine the optimal window size and its impact on albedo estimates. Performance measures and evaluation criteria to select the optimal spatial window size and evaluate the albedo validation are examined. Ground measurements of albedo from Arctic and alpine glaciers and ice caps were consolidated and used for validating the HSA outside the GrIS. Finally, the cross sensor difference between the latest L9 OLI-2 and L8 OLI datasets over snow/ice covered areas was evaluated for the first time, and L9 was added into the HSA product.

## 2. Method

The workflow is outlined in Figure 2. It consists of four main steps: 1) HSA data processing; 2) cross sensor analysis to add the L9 OLI-2 to the HSA dataset; 3) spatial window size analysis to determine the optimal scale for validation; and 4) albedo validation at different arctic and alpine sites. The performance measures and evaluation criteria are also discussed in this section.

### 2.1. Harmonised satellite albedo data processing

Shortwave broadband albedo (SBA, in the wavelength range of 400–2,500 nm), presented here, approximates broadband albedo in glaciological remote sensing (Cogley et al. 2011; Lucht et al. 2000; Naegeli et al. 2017). The HSA ($\alpha_{\mathrm{sat}}$) uses Landsat (Collection 2, Level-2, Tier 1) and Sentinel 2 (Collection 1, Level-2A) surface reflectance (SR) products, where SR refers to the hemispherical-directional reflectance (HDRF) (Schaepman-Strub et al. 2006; van der Werff and van der Meer 2016). Images with high solar zenith angles (SZA>76°) are excluded from the HSA (Feng et al. 2023). These data were processed by following the procedures in Feng et al. (2023), and a brief summary is given below. Clouds and cloud shadows were masked using Fmask (Zhu and Woodcock 2012, 2014) and Sen2Cor (Main-Knorn et al. 2017). It is a known issue that Landsat Collection 2 SR values may be >1 due to failure in the Aerosol Optical Thickness inversion over bright surfaces (e.g. snow and ice) (Crawford et al. 2023) and ‘overcorrection’ associated with incorrect atmospheric characterization (Roy et al. 2014). These invalid SR values were masked out during the processing (Crawford et al. 2023; Feng et al. 2023). Data harmonization was conducted by the use of sensor transformation functions. The narrow to broadband algorithm (Equation.1) utilizes the visible and near infrared (VNIR or visnir) bands (Figure 1b) in the harmonized Landsat 4–8 and Sentinel 2 SR products.

(1) $$\alpha_{\mathrm{sat}}=0.7963\cdot R_{\mathrm{blue}}+2.2724\cdot R_{\mathrm{green}}-3.8252\cdot R_{\mathrm{red}}+1.4343\cdot R_{\mathrm{nir}}+0.2503$$

Modifications to the HSA have been made to adapt to a few issues concerning the utilized SR products. Landsat 7 (L7) datasets acquired since 2021 were discarded due to the impact of orbit drift on data quality (Qiu et al. 2021). The European Space Agency (ESA) recently deployed a new processing baseline PB-04.00 for both the Sentinel 2 (S2) Level-1C and Level-2A datasets acquired after 25 January 2022, which shifts the range of the digital number (DN) by 1000 (European Space Agency 2022a). A harmonized S2 MultiSpectral Instrument (MSI) dataset is made available by the Google Earth Engine (GEE) to keep the consistency of the newly processed S2 imagery with the older scenes (Google Earth Engine 2022). Therefore, the harmonized S2 SR image collection (COPERNICUS/S2 SR HARMONIZED in GEE) was utilized instead of the uncorrected S2 SR image collection (COPERNICUS/S2 SR).

### 2.2. Cross sensor analysis of Landsat 9 and 8

The HSA utilizes L8 as the reference dataset and calibrates L4–7 and S2 surface reflectance (SR) datasets to L8, and the cross sensor calibration coefficient was derived using images covering the western GrIS (Feng et al. 2023). Hence, the study area is the same as in the previous research both to keep consistency and to ensure the results are comparable. Cross sensor differences between the L9 OLI-2 and L8 OLI SR datasets were analysed to harmonize the L9 dataset.

Both the L9 and L8 SR products are available in the GEE catalogue. Bands of interest are: blue, green, red, near-infrared (NIR), and shortwave-infrared (SWIR) 1 and 2 (Figure 1b). The relationship between the reference L8 SR and L9 SR was analysed by following the procedures in Feng et al. (2023), adapted from Roy et al. (2016). All the available L9 and L8 SR images covering the Western GrIS acquired during May–August 2022 were imported into GEE. The L9 ($R_{\mathrm{band}}^{SR}$) images acquired on the same day were mosaiced and paired with L8 ($R_{\mathrm{band}}^{SR\mathrm{ref}}$) imagery captured within 24 hours (h). A modified noise filter (Equation.2) was used to mask out pixel pairs with value differences greater than the average of paired pixels. The image pairs were resampled to 600 m, and the extracted pixel values of each spectral band were statistically investigated. The band to band regressions (ordinary least square regression model – OLS; reduced major axis model – RMA) reveal the relationship between the reference L8 SR and L9 over snow/ice covered surfaces.

(2) $$\frac{|R_{b\mathrm{and}}^{SR}-R_{b\mathrm{and}}^{S\mathrm{Rref}}|}{0.5|R_{b\mathrm{and}}^{SR}+R_{b\mathrm{and}}^{S\mathrm{Rref}}|}<1$$

### 2.3. Performance measures and evaluation criteria

Various methods are available and have been recommended for model performance evaluation. The Pearson correlation coefficient (R) and the coefficient of determination ($R^{2}$) are widely used as a benchmark in model validation (Moriasi et al. 2015) and albedo product evaluation (Stroeve, Box, and Haran 2006; Wright et al. 2014), along with the root mean squared error (RMSE). However, correlation-based performance measures are sensitive to outliers but are insensitive to systematic over- or under-estimation (Krause, Boyle, and Bäse 2005; Legates and McCabe 1999). Krause, Boyle, and Bäse (2005) recommended always reporting the gradient and the intercept in addition to R and $R^{2}$. A good agreement has a slope close to one and an intercept that is close to zero. The Nash-Sutcliffe efficiency coefficient (NSE, Equation.3), proposed by Nash and Sutcliffe (1970), is a popular index to quantify the simulation performance in hydrological time series studies. The NSE ranges from −∞ to 1, where NSE=1 indicates a perfect fit and NSE<0 suggests that the mean of the observations is a better predictor than the model (Legates and McCabe 1999; Nash and Sutcliffe 1970). The index of agreement, d (Eq.4), was developed to improve on correlation-based measures (Willmott 1981; 1981, 1984). d is similar to $R^{2}$ and ranges from 0 (no agreement) to 1 (perfect fit) for the comparison of observations and predictions (Krause, Boyle, and Bäse 2005; Legates and McCabe 1999). It is more sensitive to differences between measured and model predicted means and variances (Legates and McCabe 1999; Willmott 1984) than is either $R^{2}$ or NSE.

(3) $$\mathrm{NSE}=1-\frac{\sum_{i=1}^{n}{\left(\alpha_{i}^{aws}-\alpha_{i}^{sat}\right)}^{2}}{\sum_{i=1}^{n}{\left(\alpha_{i}^{aws}-{\overset{ˉ}{\alpha }}^{\mathrm{aws}}\right)}^{2}}$$

(4) $$d=1-\frac{\sum_{i=1}^{n}{\left(\alpha_{i}^{aws}-\alpha_{i}^{sat}\right)}^{2}}{\sum_{i=1}^{n}{\left(|\alpha_{i}^{sat}-{\overset{ˉ}{\alpha }}^{\mathrm{aws}}|+|\alpha_{i}^{aws}-{\overset{ˉ}{\alpha }}^{\mathrm{aws}}|\right)}^{2}}$$

Performance measures that quantify the difference between observations and model predictions as squared values tend to overestimate the differences associated with large measured values and underestimate those of the low values (Krause, Boyle, and Bäse 2005; Legates and McCabe 1999). Typically, the albedo of fresh dry snow is 0.85 or higher, while that of ice varies between 0.20 and 0.65 (Cuffey and Paterson 2010). Snow errors will tend to bias the regression fits as a consequence. One of the modified forms of NSE uses logarithm (lnE) of observations ($\ln\alpha_{i}^{aws}$) and predictions ($\ln\alpha_{i}^{sat}$) and is thus a popular way of reducing the problem of the squared differences (Krause, Boyle, and Bäse 2005; Moriasi et al. 2015). The other modified Nash-Sutcliffe efficiency coefficient ($E_{j},\;j\in N$, Equation.5) can significantly reduce the over-sensitivity to extreme high values and is more sensitive to low values (Krause, Boyle, and Bäse 2005). The sensitivity of $E_{j}$ to high values increases as the j increases; therefore, j=1 is used in the evaluation of ice albedo validation.

(5) $$E_{j}=1-\frac{\sum_{i=1}^{n}|\alpha_{i}^{aws}-\alpha_{i}^{sat}{|}^{j}}{\sum_{i=1}^{n}|\alpha_{i}^{aws}-{\overset{ˉ}{\alpha }}^{\mathrm{aws}}{|}^{j}}$$

Each of the performance measures has its own advantages and disadvantages, and evaluation criteria can be determined both statistically and graphically (Moriasi et al. 2015). In this paper, the optimal spatial window size (sec.2.4) is chosen by maximizing the statistical measures ($R,R^{2},\mathrm{NSE},\mathrm{lnE},E_{j}$) and minimizing the RMSE and bias. The albedo quality (sec.2.5) is assessed using the statistical measures and graphical measures which provide supplementary evidence (i.e. slope and intercept of the linear best fit line, distribution of data).

### 2.4. Spatial window size

The optimal spatial window size is the scale that best represents the geographical point of interest (Marceau et al. 1994; Wu et al. 2019). The validation of albedo is a point-to-pixel process (Figure A1) that requires geolocation of the AWS measurement to intersect the pixel of interest within the remote sensing data. However, the AWS measurements may only record a stationary coordinate on the day of installation or when the station was last maintained or visited. Hence, the optimal window size in the albedo validation should be able to capture the drift of the AWS with ice flow (Figure A1) and account for image co-registration error (Figure A1) without compromising the spatial resolution.

The spatial window size can be defined either arbitrarily or quantitatively (Wu and Li 2009). The fixed odd-numbered squared kernels (n×n), e.g. 3×3 (Kennedy, Yang, and Cohen 2010; Wulder et al. 2021) or 5×5 (Dai et al. 2018) or larger (Scambos et al. 1992), are commonly used when reducing the neighbouring pixels within the sampling grids to its arithmetic mean/median (Marceau et al. 1994; Paul et al. 2017). The geographic window size can also be determined statistically by the correlation of pixel values surrounding its central point of interest using semivariograms (Diehl et al. 2002; Ryan et al. 2017; Van der Meer 2012).

Landsat and S2 datasets have different spatial resolutions. Therefore, the spatial window size (n, denoting the number of pixels) does not correspond to a uniform ground sampling distance across sensors. The narrow to broadband algorithm (Equation 1) utilizes the VNIR bands, which have resolutions of 30 m for Landsat and 10 m for S2. Herein, all the units (n) of the spatial window size are converted to length scales in metres (e.g. the 90 m scale spatial window equals 3×3 pixels on Landsat and 9×9 pixels on S2).

The GrIS is an ideal target for assessing the spatial window size on ice homogeneity and for validation with *in situ* data because it has extensive AWSs measurements on its relatively flat and homogeneous surface and less cloud cover than other Arctic areas (Stroeve, Box, and Haran 2006). It has been widely used for albedo validation (Klein and Stroeve 2002; Kokhanovsky et al. 2020; Stroeve et al. 2013; Wehrlé et al. 2021). Different sizes of spatial windows are defined by progressively aggregating the images from finer to coarser scales. The impact of the changing scales of spatial windows on the homogeneity of ice surface and the performance of the albedo estimation algorithm was investigated as follows.

#### 2.4.1. Ice surface homogeneity at K-transect

The homogeneity of remote sensing imagery is affected by surface features, spatial resolution, and the scales of the spatial window (Marceau et al. 1994; Paul et al. 2017). The grey-level co-occurrence matrix (GLCM), also known as the spatial grey-level dependence matrix (SGLDM), is an approach widely used to quantitatively characterize the image texture (Conners, Trivedi, and Harlow 1984; Haralick, Dinstein, and Shanmugam 1973). It derives the texture metrics by tabulating the frequency of pairs of neighbouring pixel values in a given direction and distance (Davies 2018; Soh and Tsatsoulis 1999). The homogeneity statistics derived from GLCM enable us to examine how homogeneous the remote sensing image is (Champion et al. 2014; Soh and Tsatsoulis 1999). Values range from 0 (perfect heterogeneous) to 1 (perfect homogeneous).

**Figure 3.**
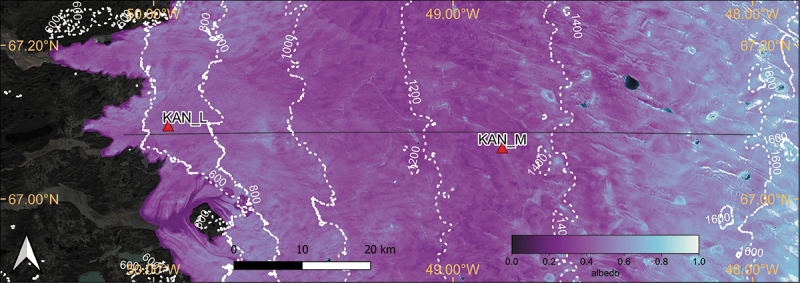
Map of harmonised satellite albedo (19–23 July 2020) at the K-transect (black line) and the two PROMICE AWSs (KAN_M and KAN_L). The contour lines are derived from the ArcticDEM (Porter et al. 2018) and shown only for areas above 400 m a.S.l.

**Figure 4.**
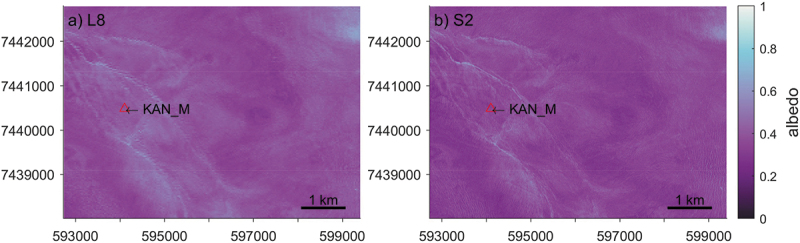
The harmonised satellite albedo derived from Landsat 8 (160×223 pixels, subfigure a) and Sentinel 2 (479×669 pixels, subfigure b) at KAN_M PROMICE AWS. The images were acquired on the same day (21 July 2020). The map is projected in WGS 84/UTM zone 22N and the location of KAN_M is labeled on the map.

The investigation started at the local scale by calculating the homogeneity at the site of PROMICE AWS KAN_M near the K-transect (Figure 3) on the GrIS. Two scenes of satellite images covering the KAN_M PROMICE AWS were acquired on the same day (21 July 2020) by L8 (Figure 4a) and S2 (Figure 4b) respectively. The S2 imagery was harmonized to L8, and the albedo was calculated by Equation.1. The L8 albedo image is 160 by 223 pixels at 30 m resolution. The 10 m resolution of the S2 albedo image was resampled to 30 m using bilinear interpolation in order to match L8’s footprint. It also allows us to evaluate the impact of spatial resolution on the homogeneity of remote sensing imagery. The homogeneity of the albedo images was assessed by the homogeneity property derived from the GLCMs of the L8, S2, and resampled S2 images.

**Figure A2.**
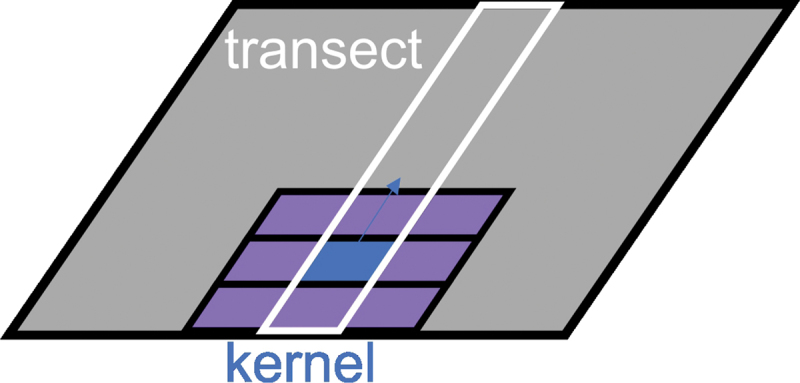
Illustration of the moving spatial window along the K-transect. The kernel sizes are 30 m, 50 m, 90 m and 150 m respectively.

The analysis of geographic variance is also a good indicator of image homogeneity and a measure of the optimal resolution (Marceau et al. 1994; Ozkan and Demirel 2021; Wu and Li 2009). The K-transect lies on the western GrIS at 67.08°N with an average equilibrium altitude of 1,553 m a.s.l (van de Wal et al. 2012) and travels through the Dark Zone (Knap, Reijmer, and Oerlemans 1996; Ryan et al. 2018; Wientjes and Oerlemans 2010) on the GrIS. A moving spatial standard deviation (SD) window (Figure A2) was applied to the HSA along a subset of the K-transect (50.1°W − 48°W, 500–1,600 m a.s.l, Figure 3) from May to September in 2019–2021. The L7 dataset was excluded to avoid contamination by pixels affected by the scan line error. The SDs of the pixels inside the spatial windows were reduced to the centre pixel along the transect at 10 m or 30 m increments for S2 and L8, respectively. The homogeneity of ice surface also varies seasonally because of evolution in the surface morphology during the melt season (Ryan et al. 2017). Transect analysis allows us to identify trends in spatial homogeneity during the melt season. We note that the evolution of the surface morphology during the melt season (Ryan et al. 2017) also impacts the homogeneity of the ice sheet surface.

#### 2.4.2. Window size and algorithm performance

**Figure 5.**
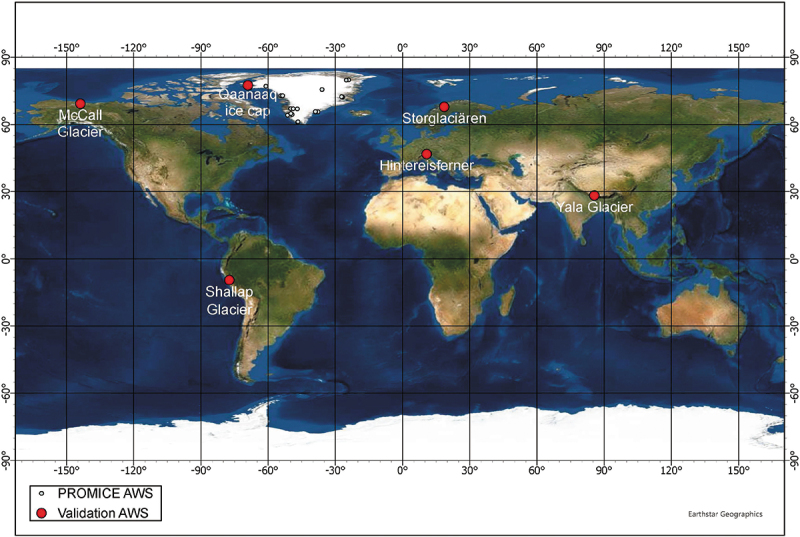
Location of the automatic weather station (AWS) sites used for global *in situ* validation. Further information on each site is given in Table B2. In total, 31 (PROMICE: 25, other: 6) AWS sites are used. The basemap is provided by ESRI, Earthstar Geographics.

The spatial window size affects the agreement between the predicted albedo and the *in situ* AWS albedometer measurements (Ryan et al. 2017). The optimal spatial window size for validating the HSA was sought, as well as an assessment of the errors arising from the use of non-optimal window size, by employing the performance measures and evaluation criteria outlined above. HSA at the locations of the PROMICE AWS (Figure 5) was extracted at scales ranging from 10 m to 150 m. Landsat derived albedo was excluded at 10 m and 20 m scales since the image resolution (30 m) is coarser than the scales of interest. The images were resampled for even-sized square windows (i.e. 20 m for S2 and 60 m scales for Landsat).

The Brown–Forsythe test (Brown and Forsythe 1974) is widely used in testing whether the input samples’ variances are equal. It was utilized to determine if the HSA data at different scales are of equal variance. The null hypothesis of equal variance assumes the differences in the window size have no significant impact on the homogeneity of pixels (Helder, Basnet, and Morstad 2010).

### 2.5. Global in situ albedo validation

The albedo validation was conducted by linearly correlating the extracted HSA with the globally collected *in situ* AWS (Figure 5) albedo measurements. Both Storglaciären in Scandinavia and Hintereisferner in the Alps are reference glaciers used by the World Glacier Monitoring Service (WGMS, https://wgms.ch/products_ref_glaciers/). The other four sites cover North and South America, Greenland, and High Mountain Asia.

The AWS measurements are treated as groundtruth and their sources are listed in the Appendix Table B2. The selected AWSs provide hourly or higher temporal resolution *in situ* meteorological records. The timestamps for the AWS albedo measurements were all converted to Coordinated Universal Time (UTC). The AWS albedo was calculated as the ratio of the total reflected to the total incoming shortwave solar radiation (Cogley et al. 2011). Invalid values (α<0 or >1) and unreliable measurements, due to tilting of the mast or to condensation reported in the raw AWS measurements, were filtered out. The consequent filtered albedo dataset was smoothed with a moving average filter with a 5-hour time window ($h_{0}\pm 2h$). PROMICE AWS data are well calibrated with high quality control; therefore, the moving average filter was not applied to PROMICE AWS albedo. The HSA was extracted at the AWS locations using the optimal scale determined in the previous step. The satellite-derived albedo was matched with groundtruth albedos that were recorded within an hour (dT< 1 h) of each other.

## 3. Results

### 3.1. Band to band regression between L9 and L8

**Figure 6.**
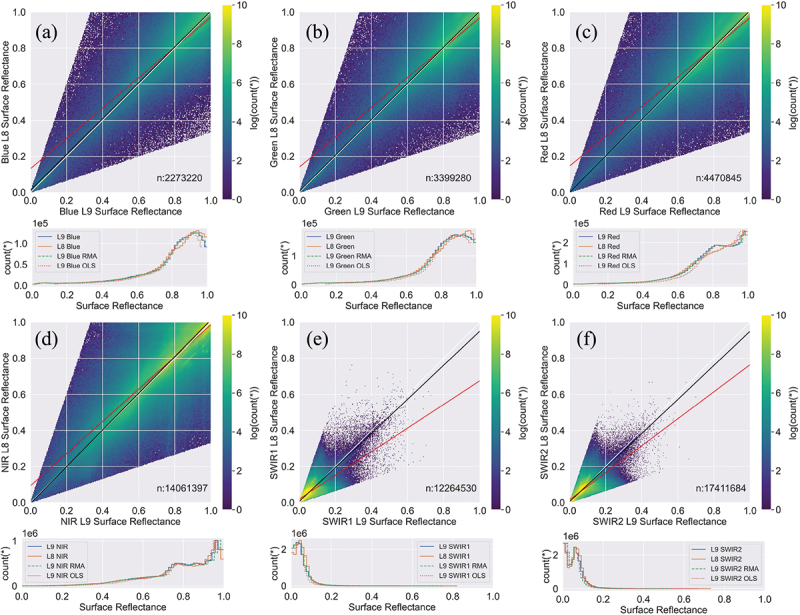
Band to band scatterplots of paired Landsat 9 OLI-2 surface reflectance against Landsat 8 OLI surface reflectance. Spectral bands are labelled in each subfigure: (a) blue, (b) green, (c) red, (d) NIR, (e) SWIR1 and (f) SWIR2. All the paired pixels were acquired during the melt season (May–September) of 2022 on the western GrIS and were resampled to 600 m resolution. Both OLS regression model (OLS: red line) and RMA model (RMA: black line) were utilized to compare the cross sensor difference between L9 and L8. The 1:1 reference line is drawn in white. The number of paired pixels (n) of each selected spectral band is marked in the scatterplots, and the colourbar range is the log-transformed number of paired pixels. The corresponding histograms of the paired pixel values and the calibrated L9 OLI-2 surface reflectance using OLS and RMA regressions are plotted in the panels below the respective scatterplots.

The cross sensor comparison of the paired L9 and L8 SR pixel values is displayed graphically in Figure 6 and summarized statistically in Table B1. The triangle-shaped data clouds in all spectral bands are the result of the noise filter (Equation 2) that masks out pixel pairs with value differences greater than their average (Feng et al. 2023). The data points reside symmetrically along the 1:1 reference line (white line). The number of paired pixels (n) in each subplot varies because different numbers of pixels are removed by the pixel saturation mask. The spectral bands of L9 are all linearly correlated with the corresponding bands of L8 (R>0.69,p<0.0001, Table B1). For the visible and near-infrared (VNIR or visnir), the slopes of the RMA models are within the range of 1±0.02, and the intercepts are very close to zero. The correlations for SWIR1 (*R* = 0.69) and SWRI2 (*R* = 0.79) bands are weaker than the VNIR bands (R>0.82). The step curves of histograms in (Fig. 6) match with each other well, indicating the cross sensor differences are minor (mean difference <0.01, Table B1). The RMA transformed L9 matches the step curve of L8 better in general.

### 3.2. Optimal spatial window size

#### 3.2.1. Ice homogeneity at KAN_M PROMICE AWS

**Figure 7.**
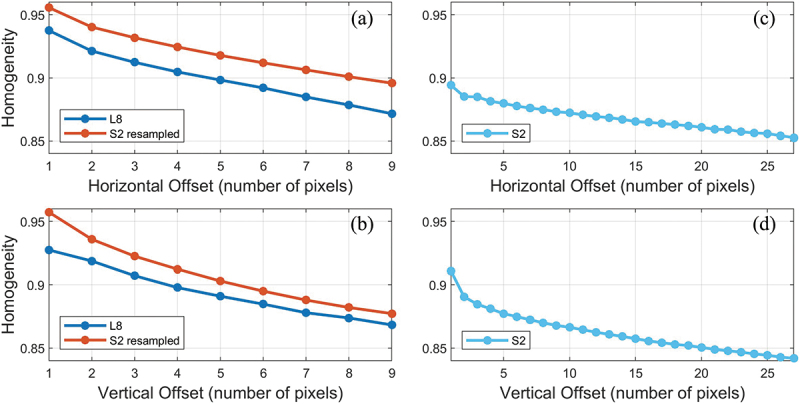
The homogeneity of the harmonised satellite albedo imagery at KAN_M PROMICE AWS (Figure 4). The image texture homogeneity is derived from the grey-level co-occurrence matrix of the albedo images as a function of horizontal (a, c) and vertical (b, d) offsets. The pixel offsets of the S2 albedo are three times that of the 30 m scale L8 and resampled S2 albedo because the Sentinel 2 image was resampled to 30 m to match the resolution of Landsat 8.

The local scale analysis shows the influence of the spatial window scale on the homogeneity of remote sensing imagery. The homogeneity of the images was plotted as a function of both horizontal (Figure 7a-c) and vertical (Figure 7b-d) offsets in the number of pixels. Here, the range of horizontal and vertical offset of the S2 albedo is three times that of the 30 m scale L8 and resampled S2 albedo due to the difference in spatial resolution. The dark ice (α<0.4 on average) surface is relatively homogeneous with GLCM homogeneity scores > 0.8. The homogeneity statistics gradually decreased as the offsets increased, suggesting that the albedo image becomes more heterogeneous as the distance between the pixel of interest and the number of its neighbouring pixels increases. On average, the homogeneity of the S2 resampled albedo image is 0.03 higher than the L8 albedo image. The two-sample t-test rejects the equal mean null hypothesis of L8 and S2 resampled albedo at the 5% significance level. The higher spatial resolution of the S2 albedo image resulted in lower homogeneity per offset compared to the 30 m resolution albedo images.

#### 3.2.2. Ice surface homogeneity at K-transect

**Figure 8.**
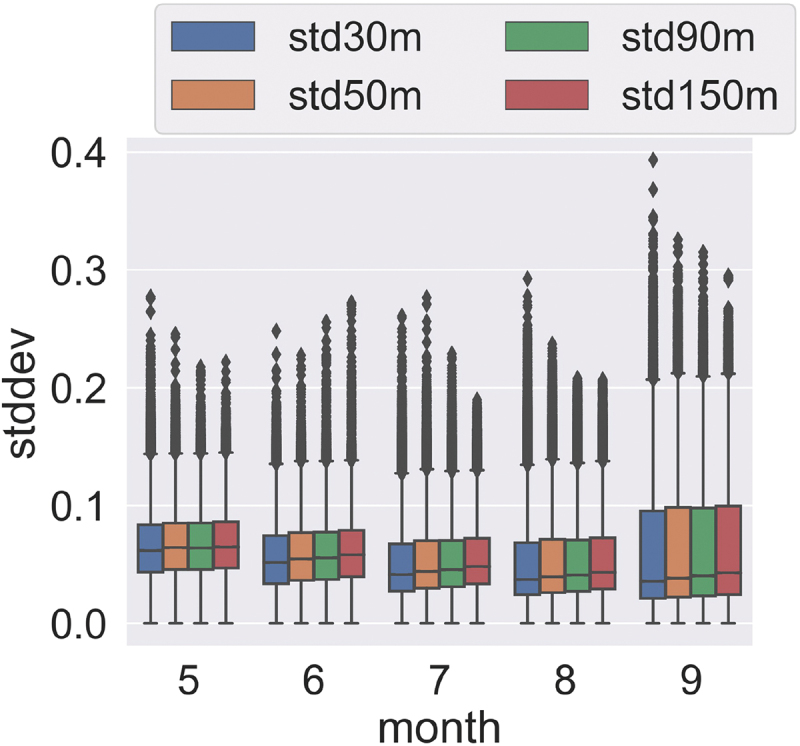
Boxplots of the standard deviation of harmonised satellite albedo along the K-transect at different scales (30–150 m) between May and September (2019–2021).

The K-transect analysis focuses on the seasonal evolution of ice surfaces and the subsequent impacts on the ice surface homogeneity. The SDs along the K-transect were grouped by month and are shown in Figure 8. The squared kernel size ranges from 30 m to 150 m (Figure A2) and Landsat data was excluded from the scales of 30 m and 50 m due to its pixel size limitation. The size of the spatial window has an impact on the pixel variances and the influence varies seasonally. Generally, smaller window sizes have lower variance. The median of the SDs lowers as the melt progresses from May to August. The transition from the melt to the accumulation season in September results in a broader range of SDs because of the impact of fresh snowfall and subsequent localized melting (Ryan et al. 2017).

#### 3.2.3. Algorithm performance with varying spatial window size at PROMICE AWSs

**Figure 9.**
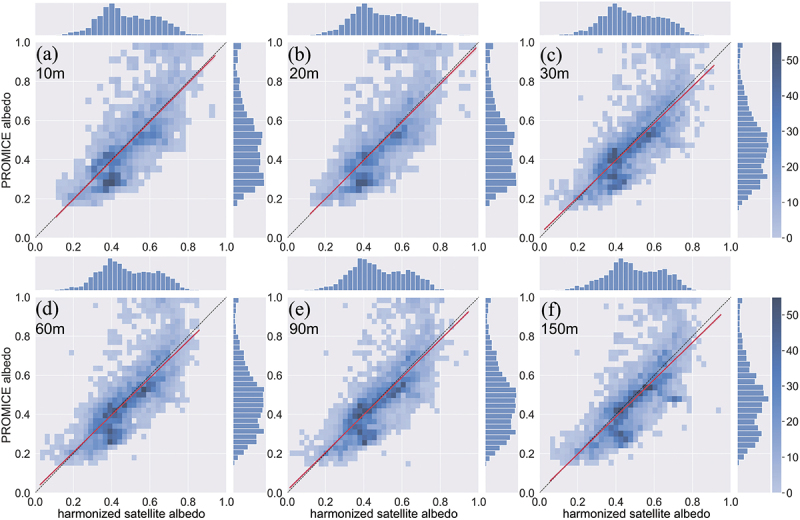
Linear regressions and 2d-histograms of harmonised satellite albedo and PROMICE AWS albedo at different harmonised satellite albedo scales, as shown in the subfigures. Landsat dataset was excluded at scales of 10 m and 20 m for the ground sampling distance is smaller than its spatial resolution (30 m). The best fit line is illustrated as the red line, and the black dotted line is the reference 1:1 line. The linear models, the correlation coefficients, and other selected statistical measures are summarized in Table 1.

The influence of the spatial window size on the albedo validation was examined statistically (Table 1) and graphically (Figure 9) by analysing the association between the HSA and the PROMICE AWS albedo measurements. Generally, the shapes of the 2d-histograms are strikingly similar regardless of the scales. The high-frequency albedo pairs reside along the 1:1 reference line (black dotted line). Outliers cluster when $\alpha_{\mathrm{aws}}>0.9$, in line with our previous work (Feng et al. 2023). Both albedo datasets have a broad bimodal distribution, as illustrated in Figure 9, and the ground AWS albedo measurements have a longer tail due to the high values of the outliers.

**Table 1.** Relationship between the harmonized satellite albedo and PROMICE AWS albedo. Scales are converted into meters based on the different spatial window sizes of Landsat and Sentinel 2. The correlation coefficient (R), slope and standard error, and intercept are summarized. All p-values are < 0.001. Also shown are the mean bias ($\alpha_{\mathrm{sat}}-\alpha_{\mathrm{aws}}$), RMSE, NSE, d, lnE, and $E_{j}$ between the harmonized satellite albedo and the PROMICE AWS albedo. Landsat data were excluded at scales of 10 m, and 20 m for the ground sampling distance is smaller than its spatial resolution (30 m).

Scale(m)	R	Slope	Intercept	RMSE	Bias	NSE	d	lnE	$E_{j}$
10	0.76	0.9919 ± 0.0155	−0.0019	0.1220	0.0059	0.5835	0.8541	0.5889	0.3866
20	0.75	0.9786 ± 0.0162	0.0045	0.1254	0.0059	0.5568	0.8428	0.5669	0.3728
30	0.79	0.9455 ± 0.0103	0.0186	0.1081	0.0078	0.6183	0.8760	0.6095	0.4207
60	0.79	0.9491 ± 0.0104	0.0168	0.1078	0.0078	0.6168	0.8750	0.6022	0.4207
90	0.79	0.9616 ± 0.0105	0.0133	0.1076	0.0051	0.6227	0.8761	0.6079	0.4318
150	0.77	0.9512 ± 0.0118	0.0075	0.1159	0.0167	0.5751	0.8566	0.5832	0.3919

The HSA data extracted at different scales were all linearly correlated with the PROMICE albedo. The performance measures, including the Pearson correlation coefficient (R), slope and intercept of the linear fit, RMSE, mean bias, NSE, d, lnE and $E_{j}$, are summarized in Table 1. The differences among the statistical performance measures at different scales are minor. The *R* values range from 0.75 to 0.79 and are significant (p-value<0.001). The NSE, d, lnE, and $E_{j}$ resemble the R values but with different explanatory powers. The slopes of the linear regression are all close to unity (slope >0.97) at 10 m and 20 m scales (Figure 9a,b, Table 1). The agreement between the HSA resampled 20 m scale and *in situ* albedo is not as good as the 10 m scales, though has the same RMSE. The derived HSA at scales between 30 and 150 m (Figure 9c-f) combined both Landsat and S2 datasets. Near perfect albedo predictions should have a slope close to 1 and maximize statistical measures, while minimizing the RMSE and bias. The 90 m scale has the highest NSE, d, lnE,$E_{j}$, and R values (Table 1). The gradient reaches its maxima (slope = 0.9616) at 90 m scale, and both the RMSE and bias are minimized as well. Hence, the 90 m scale was chosen as the optimal spatial window size.

**Figure A3.**
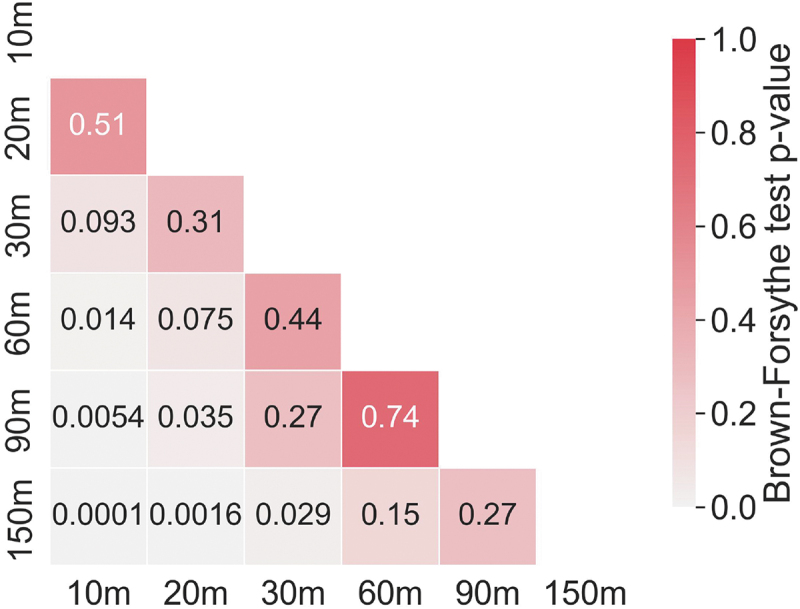
Matrix of the p-values of the Brown-Forsythe test between different scales (m) of the harmonized albedo. All the albedo values are extracted at the location of the PROMICE AWSs.

The varying spatial window size affects not only the bias between the albedo observations and predictions, but also the variance. The variances of the satellite-derived albedo and the PROMICE AWS albedo were statistically compared and analysed. The HSA and PROMICE albedo do not have equal variances, given the long tail of the PROMICE albedos (Figure 9) at higher values (α>0.9) and the Brown–Forsythe test results (p-value<0.001). The test results between HSA at different scales vary depending on the window size (Figure A3). The test for equal variances between the 60 m and 90 m scales has the highest p-value (0.74), indicating that the Brown–Forsythe test does not reject the null hypothesis. The p-value decreases as the spatial window increases or decreases from 60 m-90 m. The 10 m and 20 m scales of HSA are of equal variances (p-value>0.5) as the differences in the spatial window size are small. The HSA generally does not have equal variances if the spatial window size difference is larger than 30 m.

### 3.3. Global validation of the harmonised satellite albedo product with in situ AWS data

**Figure 10.**
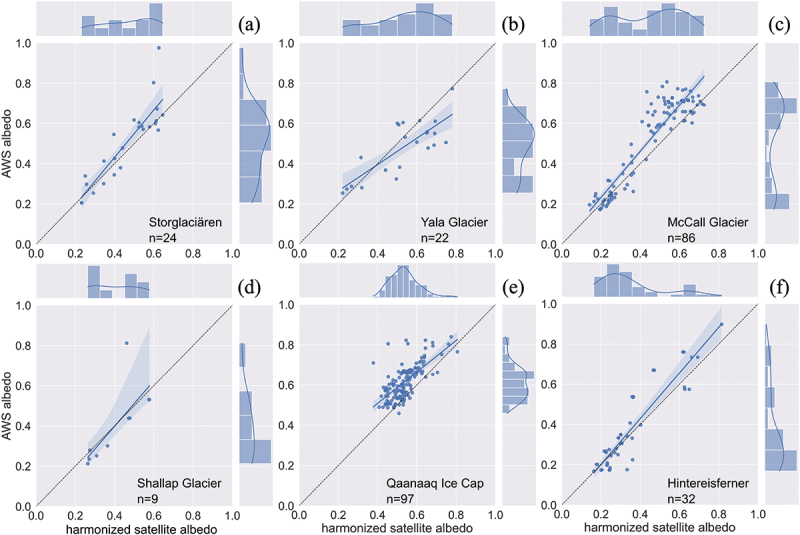
Results of the harmonized satellite albedo validation for different arctic and alpine sites: a) Storglaciären, Sweden, Scandinavia (2013–2018); b) Yala Glacier, Nepal, high Mountain Asia (2016–2019); c) McCall Glacier, Alaska, North America (2004–2014); d) Shallap Glacier, Peru, South America (2010–2012); e) Qaanaaq ice Cap, Greenland (2012–2020); f) Hintereisferner, Austria, Alps (2014–2018). The locations of these AWS sites are shown in Fig.5.

The validation results are shown both graphically (Figure 10) and statistically (Table 2). Generally, the HSA data are linearly correlated with *in situ* AWS albedo measurements at Storglaciären, McCall Glacier, Shallap Glacier, and Hintereisferner. Outliers are mostly found at higher AWS albedo ($\alpha_{\mathrm{aws}}>0.8$), which is in line with Feng et al. (2023). The deviations of slopes from 1 are less than 0.18 at all four sites and the intercepts are close to zero. Larger discrepancies between the linear best fit (blue line) and the 1:1 reference line (black dotted line) are found for Yala Glacier (slope = 0.6543, Table 2) and Qaanaaq Ice Cap (slope = 0.7839, Table 2).

**Table 2.** Statistics for the correspondence of harmonized satellite albedo vs. The *in situ* AWS albedo measurements.

Site	Region	Slope	Intercept	R(P>0.05)	NSE	lnE	d	$E_{j}$
Storglaciären	Scandinavia	1.1787	−0.0403	0.88	0.7062	0.8118	0.9006	0.5626
Yala Glacier	High Mountain Asia	0.6543	0.1338	0.79	0.3208	0.5031	0.8520	0.1928
Shallap Glacier	South America	1.1306	−0.0542	0.75	0.5536	0.7159	0.8164	0.5056
McCall Glacier	North America	1.1476	0.0024	0.92	0.7239	0.8243	0.9166	0.5639
Qaanaaq Ice Cap	Greenland	0.7839	0.1946	0.68	−0.4078	−0.6523	0.6868	−0.1708
Hintereisferner	European Alps	1.1366	−0.0260	0.92	0.8209	0.8031	0.9435	0.6295

Different performance measures suggest different conclusions for the albedo validation. The NSE and its modified form (lnE and $E_{j}$) outperform R and d in the validation of albedo. Both R and $R^{2}$ suffer from the additive and proportional discrepancies between the *in situ* albedo and the satellite-derived albedo. The AWS located on Yala Glacier in High Mountain Asia was drifting with the ice flow, but the velocity was not provided in the metadata (Gurung 2021). Therefore, the stationary coordinates in the datasheet were used instead. The high R (*R* = 0.79) and d values (d>0.85) indicate a good correlation, but the HSA is not a good predictor as the slope of the linear best fit is 0.65 at Yala Glacier. The low values of NSE,lnE, and $E_{j}$ also prove that the goodness of fit of the HSA at Yala Glacier and Qaanaaq Ice Cap is not as good as at the other four AWS sites. The SIGMA-B AWS at Qaanaaq Ice Cap was installed at an elevation of 944 m a.s.l (Nishimura et al. 2021), which is slightly above the equilibrium line altitude of 916 m a.s.l on average between 2012 and 2015 (Aoki et al. 2014; Onuma et al. 2022; Tsutaki et al. 2017). The physical properties of the surface ice (weathering crust, superimposed ice) and the factors influencing the albedo lowering of the ice cap (Onuma et al. 2018, 2022, 2023) are significantly different from the Greenland Ice Sheet, where the darkening of surface ice is primarily driven by the growth of glacier ice algae (Anesio et al. 2017; Chevrollier et al. 2023; Cook et al. 2017, 2020; Halbach et al. 2022; Lutz et al. 2018; Ryan et al. 2018; Stibal et al. 2017; Tedesco et al. 2016; Yallop et al. 2012). Frozen slush at the AWS sensor scale would appear very bright in the AWS data, but the coarser spatial resolution of the HSA would lead to smoothed albedo values. The highly heterogeneous slushy surface during the melt season may account for the poor performance of the HSA product. The SZA in high latitude regions has a greater impact on albedo products (Stroeve, Box, and Haran 2006; Stroeve et al. 2005), and may have also influenced the result. The Shallap Glacier shows a good linear correlation (Figure 10d), but the NSE value is low (<0.6) since NSE is more sensitive to high value outliers, which are present in the generally rather smaller dataset.

## 4. Discussion

### 4.1. Band to band regression of L9 vs L8

In this study, we conducted the first comparison of L9 and L8 datasets over snow/ice covered areas. L9 OLI-2 is nearly identical to L8 OLI in the vis-swir bands (Masek et al. 2020). The band to band regression (Figure 6 and Table B1) also confirms that the sensors are comparable. Data harmonization is not necessary because the cross sensor difference between L9 and L8 SR is very small (Figure 6, Table B1). The VNIR bands match more closely than the SWIR bands. This was also found in Feng et al. (2023). However, this does not impact on our HSA product as the narrow to broadband conversion does not rely on the SWIR band.

The L9 is thus added to our HSA product and extends its covered time range to today and enables a continuation of the time series into the future. Improvements to L9 OLI-2 were made regarding the spectral, radiometric non-linearity, and spatial characterization (Masek et al. 2020). It also replaces the degraded L7 dataset due to its orbital drift from 2021 (Qiu et al. 2021). The combination of L8 and the newly launched L9, calibrated and characterized for ensuring interoperability with the S2 dataset, has a revisiting time of 8 days (Wulder et al. 2022). The data harmonization will increase the data density, which is critical in time series analysis (Feng et al. 2023; Zhang et al. 2021), and facilitate remote sensing of glaciated regions with medium resolutions.

### 4.2. Performance measures and evaluation criteria

Each of the performance measures has its advantages and disadvantages. The goodness of fit of the satellite-derived product should not be assessed solely by statistical measures. The combination of statistical and performance measures is a better indicator for finding the best fit, since high values of statistical measures may be achieved despite bad model performance (e.g. Yala Glacier and Qaanaaq Ice Cap in Figure 10b). NSE is not sensitive to systematic over- or underestimation (Krause, Boyle, and Bäse 2005).

The high extreme values have a great influence on many of the performance measures (e.g. $R^{2},\mathrm{NSE},d$) due to the frequent use of squaring in statistical calculations (Krause, Boyle, and Bäse 2005; Legates and McCabe 1999). High extreme albedo values recorded by the *in situ* AWSs in the event of fresh snowfall within a 1-hour data acquisition time window (dT<1h) or small snow patches within the effective radius of the pyranometers, that cannot be detected by the coarse satellite imager are likely to hamper the validation of satellite-derived albedos. Cloud contamination of images may also create a large discrepancy between the AWS measurements and the satellite albedo product (Stroeve, Box, and Haran 2006). High *in situ* albedo outliers are found at PROMICE AWSs, Storglaciären, McCall Glacier, and Shallap Glacier (Figures 9, 10a-d). The modified form of NSE, such as lnE and $E_{j}$ (j=1), are less sensitive to high value outliers and are more susceptible to low values (Krause, Boyle, and Bäse 2005; Moriasi et al. 2015). The disadvantage of lnE and $E_{j}$ is that both may yield lower values than the statistical methods using equations with squared values, which may be wrongly interpreted as worse model performance (Krause, Boyle, and Bäse 2005). Therefore, the evaluation criteria for a good model need to be adjusted when applying lnE and $E_{j}$ as performance measures. The d statistics always produce higher values, by contrast, and it is hard to discriminate between the different model performances. The validation process should use both statistical and graphical measures and always report the slope and intercept of the linear best fit. The modified forms of NSE (i.e. lnE and $E_{j}$) are recommended when data are sensitive to outliers.

### 4.3. Spatial window size

#### 4.3.1. Ice surface homogeneity

Surface features, spatial resolution, and spatial window size can all affect the homogeneity of remote sensing images (Marceau et al. 1994; Paul et al. 2017).

Satellite images become more homogeneous with increasing spatial window size, either because the surface features have length scales that are larger than the resolution of the original satellite image and/or when the ground features become aggregated as the spatial window increases (Marceau et al. 1994). Hence, a smaller spatial window that matches the ground sampling distance of *in situ* data is recommended when an accurate real-time geolocation of the ground measurements is known, and the image co-registration error is low. The consequence of a larger spatial window scale is smoother surface features. Therefore, the resampled S2 albedo showed a higher homogeneity score than the original S2 albedo image (Figure 7a-c). However, the resampled S2 albedo also showed a higher homogeneity score than L8 albedo imagery (Figure 7a,b).

The K-transect analysis shows that the homogeneity of the surface ice varies both with change of spatial window size and season (Figure 8). Smaller spatial windows aggregate fewer neighbouring pixels and result in lower SDs. The variances of albedo along the K-transect also respond to seasonal variations. The surface ice becomes more homogeneous (lower SDs) as the melt season progresses, most likely due to the reduced area of mixed snow-ice covered pixels and the presence of water.

#### 4.3.2. Optimal spatial window size

Any selection of the window size is a compromise between the representativeness of spatial details, pixel noise, and image misregistration (Kennedy, Yang, and Cohen 2010). The optimal scale should be able to capture the moving point of interest that flows with the ice without compromising the validation accuracy. The HSA extracted at different scales does not have equal variance if the window size difference exceeds 30 m (Figure A3). The maximum footprint diameter of the mounted pyranometers at PROMICE AWSs is about 21 m under ideal installation conditions in the ablation season (Van as and Fausto 2011). However, the effective ground sampling distance of the albedometer is smaller because the cosine response and the height of the sensor above ground vary seasonally (Ryan et al. 2017). The georeferencing of the S2 image collection is 0.3 pixels for multi-temporal registration in glacier remote sensing (European Space Agency 2022b; Kääb et al. 2016), and the 10 m scale assumes that the geolocation of the sampling site lies exactly in the pixel of interest. The slope of the linear fitting is at its closest to 1 (0.99) at 10 m scale, but the bias and RMSE are highest (Table 1). The even-sized spatial window (20 m) reduced the goodness of fit due to the resampling of pixels. The 90 m scale is considered as the optimal window size as it has the highest R,NSE,d,lnE, and $E_{j}$ values, and the slope is closer to 1 compared to other scales when combining both Landsat and S2 datasets. It also minimizes the bias and RMSE as well. Other glaciological remote sensing applications may also utilize this window size or determine a bespoke optimal window size by implementing a similar analysis.

### 4.4. Albedo validation

The goodness of fit of the HSA was evaluated by comparison with *in situ* albedo measurements from AWSs in Arctic and alpine glaciers and ice caps. Generally, the HSA product performs well for the test sites (Figure 10 and Table 2) and can provide a reliable long time series of ice albedo at 30 m resolution in any area of interest.

The validation process has limitations too. The albedo validation assumes that the AWS measurements are absolute ground truth values. This assumption is prone to errors introduced by various aspects of the field instrument installation, maintenance, and data collection, particularly: 1) the lack of cross sensor validation of the AWS pyranometers; 2) the AWS site selection and the representativeness of the chosen sites; 3) the tilting of the AWS mast and the sensor’s height variation due to melt; and 4) the coordinates precision and the lack of ice drifting records, etc. The installation and maintenance of AWS are challenging in extreme environments. The impact of drifting snow height, tilting of the pyranometers during the melt season, and cloud contamination on the data are hard to identify from the AWS albedo records alone (e.g. Yala Glacier, McCall Glacier, and Shallap Glacier do not have tilting records). The lack of ice surface velocity data forced us to extract the HSA at a fixed site while the AWS was drifting away with surface ice flow (Gurung 2021). This drift from higher to lower elevation may explain some of the large deviations of the linear best fit from the 1:1 reference line (Figure 10b).

These constraints make it hard to reprocess the *in situ* data into a consistent and cross-calibrated dataset. The spatial upscaling of *in situ* data was not conducted because of the seasonal change in the ice surface features, as the homogeneity (Ryan et al. 2017) can limit the reliability of the synthesized dataset (Wu et al. 2019; Xu et al. 2018). Data cleaning is impossible as the ground albedo data are accessed from open-access sources. Hence, the 5-h moving average filter was applied to AWS data to smooth the highly noised dataset, except for PROMICE albedo measurements. The temporal smoothing may include cloud-contaminated albedo or albedo with high solar zenith angles (SZA), which influence the albedo greatly (Schaaf, Wang, and Strahler 2011; Stroeve et al. 2005; Wang and Zender 2010). However, the influence on the results should be relatively small. The HSA dataset was derived from the high-level Landsat and Sentinel 2 products, excluding data with high SZA (>76°). Hence, we are focusing on the summer albedo when the range of SZA should be stable. Snow albedo may exceed 0.9 under high SZA and optically thin clouds weather conditions (Stroeve, Box, and Haran 2006), while α>0.9 is rarely observed in our validation results (Figure 10). Wang and Zender (2010) found that the simulated albedo dependence on SZA is <0.06 as SZA increases from 0° to 90°, which is smaller than the bias caused by instrument error (Schaaf, Wang, and Strahler 2011; Stroeve et al. 2005).

## 5. Conclusions

This study focuses on two potentially important aspects of the validation of the satellite albedo derived from our harmonized Landsat and S2 datasets.

First, the influence of the spatial window size on the ice homogeneity and albedo validation was analysed both at the local scale and along the K-transect. The findings suggest that the homogeneity of the ice surface increases as the spatial resolution decreases or when the spatial window size becomes larger. The ice homogeneity also changes as the melt season progresses. The transition between the melt and accumulation seasons makes the ice surface more heterogeneous. The 90 m scale was determined as the optimal spatial window size for albedo validation.

Second, the validation shows that the HSA has good agreement with *in situ* groundtruth measurements (R ranges from 0.75 to 0.92) with AWS sites in various Arctic and alpine sites. The performance of the HSA shows that it is a reliable global ice albedo product and can serve as an essential input for change detection, surface energy budget, and ice dynamics studies in other regions worldwide. The validation was evaluated by both graphical and statistical performance measures. Statistical performance measures (e.g. lnE and $E_{j}$) that are more sensitive to low albedo values are recommended for ice albedo validation, while $R^{2}$ and NSE are more suitable when the values are high (e.g. over snow surface). We note that the data saturation issues in earlier Landsat sensors (i.e. Landsat 4–7) are not addressed in this study.

Finally, we find that the cross sensor difference between L9 OLI-2 and L8 OLI is minor and hence data harmonization is not necessary when combining L9 and L8 SR datasets. The HSA now includes L9 to enable high temporal monitoring of Arctic and alpine glaciers and ice caps.

## Data Availability

The source code for data processing and analysis is available at GitHub: https://github.com/fsn1995/Remote-Sensing-of-Albedo (doi: 10.5281/zenodo.7642574). The harmonized satellite albedo is available in a web app (https://fsn1995.users.earthengine.app/view/albedoinspector). Users can interactively obtain time series of albedo data and albedo images at ice surfaces delineated by ice masks from the Greenland Ice Sheet Mapping (GIMP) (Howat, Negrete, and Smith 2014) and the Global Land Ice Measurements from Space (GLIMS) (GLIMS Consortium 2005; Raup et al. 2007). PROMICE AWS data is accessed at https://dataverse.geus.dk/dataverse/AWS (Fausto, Van As, and Mankoff 2022). The in situ AWS data for validation are summarized in Table B2 and the satellite data are available in Google Earth Engine.

## Appendix B

**Table B1.** Summary of the cross sensor analysis results (Figure 6). The cross sensor analysis includes band to band regression models (ordinary least square, OLS, and reduced major axis, RMA), the total number of paired pixels (n), the Pearson correlation coefficient R, and the root-mean-square error (RMSE) between $R_{\mathrm{band}}^{L9}$ and $R_{\mathrm{band}}^{L8}$.

Band	Regression Type	Regression Coefficients	Paired Pixels Count (n)	R (P>0.0001)	RMSE	Mean Difference L8-L9
Blue	L9 vs L8 RMA	L8 = 0.9929 ⋅ L9 + 0.0123	2273220	0.85	0.1068	0.0067
	L9 vs L8 OLS	L8 = 0.8409 ⋅ L9 + 0.1319				
Green	L9 vs L8 RMA	L8 = 0.9979 ⋅ L9 + 0.0060	3399280	0.83	0.1032	0.0043
	L9 vs L8 OLS	L8 = 0.8284 ⋅ L9 + 0.1405				
Red	L9 vs L8 RMA	L8 = 1.0086 ⋅ L9–0.0005	4470845	0.82	0.1031	0.0063
	L9 vs L8 OLS	L8 = 0.8230 ⋅ L9 + 0.1466				
NIR	L9 vs L8 RMA	L8 = 1.0168 ⋅ L9–0.0041	14061397	0.88	0.0877	0.0091
	L9 vs L8 OLS	L8 = 0.8928 ⋅ L9 + 0.0933				
SWIR1	L9 vs L8 RMA	L8 = 0.9437 ⋅ L9 + 0.0064	12264530	0.69	0.0257	0.0037
	L9 vs L8 OLS	L8 = 0.6550 ⋅ L9 + 0.0202				
SWIR2	L9 vs L8 RMA	L8 = 0.7479 ⋅ L9 + 0.0162	17411684	0.79	0.0248	0.0018
	L9 vs L8 OLS	L8 = 0.9449 ⋅ L9 + 0.0049

**Table B2.** The list of selected AWS sites and respective source information (Figure 5).

Site	URL	Access Date	Reference
Storglaciären	Provided by Tarfala Research Station at request	2019-03-01	(Tarfala Research Station 2022)
Yala Glacier	https://doi.org/10.26066/RDS.1972507	2022-09-16	(Gurung 2021)
Shallap Glacier	https://acinn-data.uibk.ac.at/pages/shallap-glacier.html	2022-09-16	(ACINN 2022b; Gurgiser et al. 2013)
Hintereisferner	https://acinn-data.uibk.ac.at/pages/hintereisferner.html	2022-11-02	(ACINN 2022a)
McCall Glacier	https://doi.org/10.18739/A27S7HS5V	2022-09-16	(Nolan 2019; Troxler et al. 2020)
Qaanaaq Ice Cap	https://ads.nipr.ac.jp/dataset/A20220413–006	2022-10-14	(Nishimura et al. 2021)
